# A Methodology to Obtain the Accurate RVEs by a Multiscale Numerical Simulation of the 3D Braiding Process

**DOI:** 10.3390/polym14194210

**Published:** 2022-10-07

**Authors:** Wei Zhou, Hui Wang, Yizhe Chen, Yaoyao Wang

**Affiliations:** 1 Hubei Key Laboratory of Advanced Technology for Automotive Components, School of Automotive Engineering, Wuhan University of Technology, Wuhan 430070, China; 2 Hubei Collaborative Innovation Center for Automotive Components Technology, Wuhan 430070, China; 3 Hubei Longzhong Laboratory, Xiangyang 441000, China; 4 Jiangsu Xinyang New Material Co., Ltd., Yangzhou 225000, China

**Keywords:** 3D braiding composite, finite element analysis (FEA), preform, virtual fibers, representative volume element (RVE)

## Abstract

To accurately evaluate the mechanical performance of three-dimensional (3D) braiding composites, it is essential to consider the braiding process and generate realistic representative volume element (RVE) structures. An efficient simulation methodology based on truss elements was used to simulate the 3D four-directional (3D4D) braiding process utilizing the finite element method (FEM) on the macroscale. The goal was to obtain the spatial trajectories of yarns and establish the relationship between the braiding parameters and the preform structure. Based on the initial yarn topology, the yarns were discretized as bundles of virtual sub-yarns. Then, a temperature drop simulation using hybrid elements was implemented to deform the yarn cross-section and obtain the interior, surface, and corner cells on the mesoscale. The simulation results show good agreement with the experiment. A parametric study was deployed to identify the effect of the model input parameters on the computation cost and accuracy. Furthermore, the approach applies to the other braiding processes, such as the cylindrical braiding composite.

## 1. Introduction

Composite materials are an effective means of lightweight design [1]. Three-dimensional braiding composites are formed by interweaving yarns in space, and the mechanical performance in each direction can be designed by changing the braiding process [2]. In addition, a rational braiding process can produce near-net-shape products, which eliminates an additional machining process [3]. With these advantages, 3D braiding composites have significant application potential in the aerospace, marine, and transportation industries [4]. Nowadays, 3D braiding equipment is fully automated to produce composite components, which significantly increases the application and production efficiency of braiding composites.

The macro-mechanical properties of 3D braiding composites are primarily determined by the preform’s mesostructure and the constituent materials’ mechanical properties. Accurate mesostructure is a precondition for calculating the mechanical properties of composite products. The representative volume element (RVE) is the smallest volume over which a measurement can be made to yield a value representative of the whole [5]. Wang et al. [6] established three types of RVEs corresponding to the interior, surface, and corner cells, by observing the interweaving topology of yarns during the braiding process. Chen et al. [7] used an analytical approach combined with experimental observations to study the mathematical relationships between the braiding structure and the braiding process parameters. Zhang [8] analyzed the spatial motion trajectories of yarns for 3D five-directional and 3D full five-directional braiding composite materials. It was assumed that the shapes of the yarn section differed according to the compaction conditions of the yarns. Therefore, three RVEs were established by fitting the yarn space trajectory through the spline curves and connecting the sections sequentially.

The preceding studies have constructed the RVE based on many assumptions with respect to yarn geometry. For example, the yarn sections are unchangeable or are several simple sections, the yarns do not slip against each other, or the yarn path in the preform is identical to the carriers’ path in the machine bed. However, a 3D braiding fabric has a complicated structure, often with multidirectional deformation, as well as changes in the cross-sectional shape owing to yarn compaction. Therefore, many researchers have made experimental investigations to obtain accurate RVEs. Byun et al. [9] polished and observed the successive cross-sections of the composite samples in the braiding orientation, indicating that the yarn patterns of the sequential cross-sections were all anti-symmetric about the central point of sections. Then, the sequential sections of each yarn were connected to reconstruct the yarn path. In addition, the contact locations among yarns were shifted, and the cross-sectional shapes of yarns were changed in the intertwining process [9]. The precise mesostructure of the textile composite can be established using CT tomography [10,11]. Liu et al. [12] obtained the statistical data of the axial yarn characteristic parameters of the 3D five-directional (3D5D) braiding composites through a micro-CT experiment. The twisting and compressional deformation of the axial yarn was thoroughly investigated and reconstructed. Furthermore, the tracking and recognizing method for a cross-sectional shape were developed to automatically establish the geometry model of the 3D four-directional (3D4D) composite using a sequence of tomographic images [13].

It is time-consuming to use these experimental-based approaches to obtain fabric structure, which can only be used to observe product results rather than for early predictive guidance on the process. The simulation-based method is excellent for studying the process and mesostructure of a composite. Wang and Zhou [14,15] developed the digital element method, which divides a yarn into several chains of truss elements that can capture the yarn deformation produced by the braiding process. The DEA Fabric Mechanics Analyzer^TM^ software, based on the digital element method, obtained a good agreement with X-ray CT images of a 3D woven textile composite [16,17]. A comparable technique of the virtual digital elements was realized with the commercial FE codes LS-DYNA^TM^ [18] and TEXGEN^TM^ [19,20]. Green et al. [21] simulated the waving and compaction process of a 3D woven composite, and the corresponding parametric study was implemented to achieve a balance between the analysis speed and accuracy. The digital element methodology considering the rearrangement behavior of sub-yarns was used to predict the mechanical behavior of a 3D woven fabric, doing away with the need for a complicated constitutive model [22]. However, these methods do not consider the crucial effect of manufacturing process parameters on the mesostructure of the preform. The two-dimensional braiding process simulation considering the yarn as the truss element was used to study the motion parameters of the mandrel [23]. This solution does not consider the yarn squeeze deformation, which significantly affects the accuracy of the mechanical performance analysis. Ghaedsharaf et al. [24,25] modeled the geometry of biaxial braids using bundles of virtual fibers, in which the tension force was applied to the yarn tends to shrink the preform from a loose state to a tight state. However, to obtain the desired final pitch length, the initial pitch length and outer diameter, which are crucial to the final shape of the preform, were determined through a trial-and-error method [24]. Yang et al. [26] simulated the actual weaving process in an explicit solver ABAQUS/Explicit, in which each yarn was represented as several twisted sub-yarns. Nonetheless, it is computationally intensive to obtain quasi-static simulation results of the weaving process based on the virtual fibers. An efficient simulation method that considers both the braiding process and the yarn extrusion deformation, essential for obtaining the accurate RVE geometry, has not been reported so far.

This paper proposes a multiscale methodology for obtaining accurate RVEs. The study is organized as follows: Section 2 describes the 3D four-directional braiding process and the path of each yarn carrier on the machine bed. Section 3 presents the detailed implementation process of the multiscale methodology. The parametric study is conducted in Section 4. In Section 5, the cylindrical braiding process is simulated to present the method’s versatility. Concluding remarks are provided in Section 6.

## 2. Three-Dimensional Four-Directional Braiding Process

**Figure 1:**
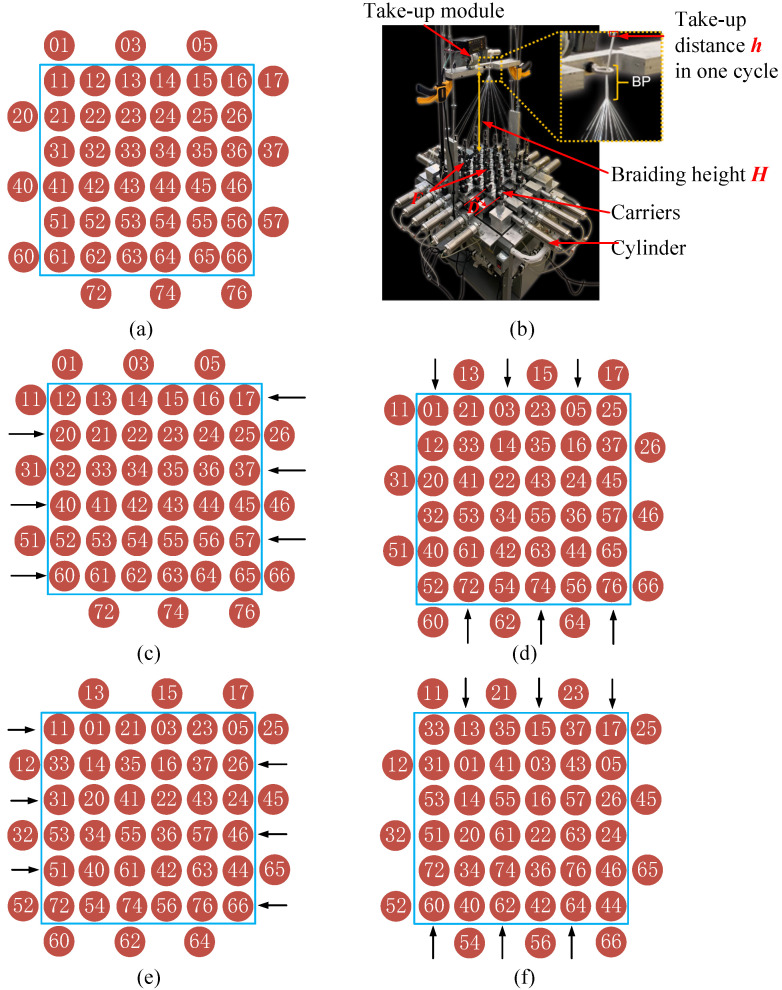
Schematic of the 3D four-directional braiding process: (**a**) the original arrangement, (**b**) the braiding machine [27]. Reprinted with permission from [27]. Copyright 2020, The Regenerative Engineering Society. (**c**) Step 1, (**d**) Step 2, (**e**) Step 3, and (**f**) Step 4.

The 3D braiding fabric is woven by the alternate movements of yarn carriers in row and column directions. Figure 1 is a schematic illustration of the four-step 1 × 1 rectangular braiding process. The arrangement of the carrier pattern (Figure 1a) determines the section shape of the preform. A machine cycle is divided into four movements: in Step 1, the yarn carriers move alternately in one position laterally (Figure 1c); in Step 2, carrier motions occur in alternate columns (Figure 1d); Steps 3 and 4 reverse the carrier movement direction in Steps 1 and 2 (Figure 1e,f), as illustrated by the arrows in each step. After these four steps, the carrier pattern is identical to the original pattern (Figure 1a). One segment of the preform is fabricated and called a braiding pitch, denoted by h. The take-up device moves up one pitch length simultaneously. The carriers regulate the tension force by retracting the yarns into the spool or releasing the yarns from the spool. Therefore, the yarns compact with each other, resulting in a stable and tight preform. The braiding process is fully automated, without a manual jamming process. Figure 1b shows an actual braiding setup, including cylinders in each line, the carriers, and the take-up module [27].

The count of carriers in the rectangle pattern is determined by the row and column number of yarns of the main part [m×n], which is given as,

(1) N=mn+m+n

where *m* and *n* are the row and column yarn numbers in the main part, respectively, and *N* is the total number of yarns. After 4N/t steps, all carriers return to the original position, where t denotes the greatest common divisor of *m* and *n*.

**Figure 2:**
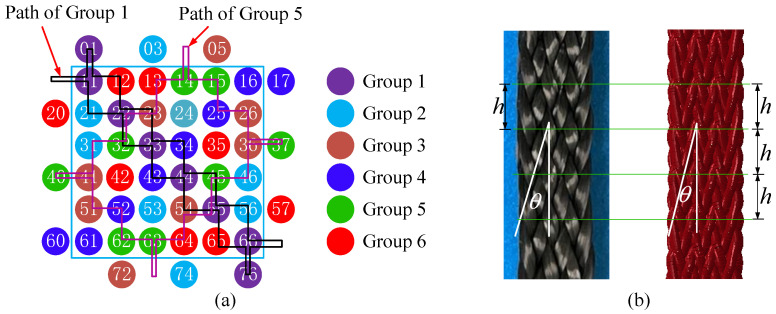
(**a**) Schematic of the yarn path and groups and (**b**) the 3D4D braid pattern of the test specimen and the simulation results.

These yarns in the [6×6] arrangement are divided into six groups according to their trajectories, as shown in Figure 2a. After moving 32 steps, all carriers return to their original positions. A Python script was written to obtain the path of every carrier and generate the boundary condition of the numerical simulation model.

Table 1 provides the main parameters of the 3D4D braiding process, as shown in Figure 1b, which significantly affects the braiding preform structure. The preform structure is mainly characterized by the interior braiding angle γ, the surface braiding angle θ, and the pitch length h. In the specimen, the surface braiding angle and the pitch length were easy to observe and measure and thus were used to benchmark with the simulation model, as shown in Figure 2b. The high-strength 12K T700 carbon yarn was used to braid the preform. The primary material properties of the yarn are listed in Table 2.

## 3. Modeling Approach Using the FEA Method

**Figure 3:**
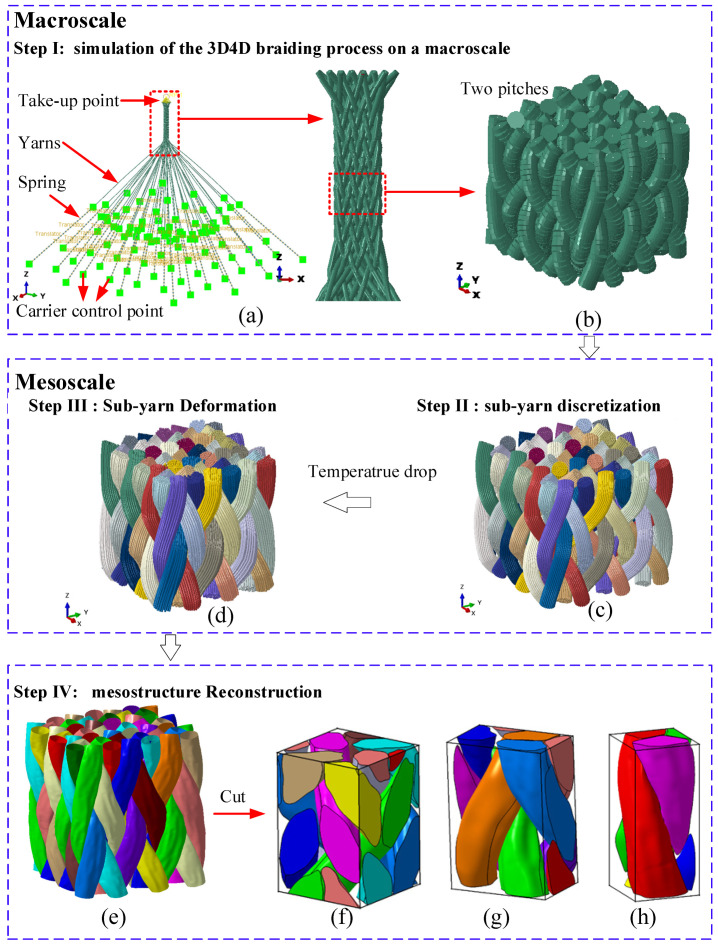
Workflow of the modeling methodology: (**a**) Step I: the simulation model of the braiding process on the macroscale, (**b**) extracting the two pitches fabric in the stable stage, (**c**) Step II: sub-yarn discretization, (**d**) Step III: sub-yarn deforming simulation on the mesoscale, (**e**) mesostructure reconstruction, (**f**) the interior cell, (**g**) the surface cell, and (**h**) the corner cell.

The workflow of the proposed modeling methodology is illustrated in Figure 3, and the procedures are described as follows:

- Simulation of the 3D4D braiding process on the macroscale: The braiding process was simulated according to Section 2, during which the yarns were discretized as T3D2 truss elements. The parametric model was generated using a Python script and solved by the Abaqus software (Figure 3a). Yarns were interweaved to achieve the preform.
- Sub-yarn discretization: The yarns in the two pitches of the preform at the stable braiding stage (Figure 3b) were divided into several bundles of sub-yarns according to the center curve of the yarns achieved in Step I, as shown in Figure 3c. A Python script was written to automate the model generation and the assignment of boundary conditions.
- Sub-yarn deforming simulation on the mesoscale: The contraction process was accomplished by imposing the temperature drop load with an appropriate thermal expansion coefficient (Figure 3d). The sub-yarns were represented by the hybrid elements consisting of the truss and beam element with coinciding nodes. Then, the yarn section changed from a circle to a realistic shape as the average stress of all sub-yarns reached the tension stress of the carrier retraction.
- Mesostructure reconstruction: Since the FEA analyses require yarns with a solid geometry rather than fibers, the chains of sub-yarns must be converted to solid geometry. Another Python script was written to extract the section shapes of different yarns and reconstruct the accurate yarn structure (Figure 3e). Eventually, three RVEs were generated: interior cell, surface cell, and corner cell (Figure 3f–g).

### 3.1. Simulation of the 3D4D Braiding Process on the Macroscale

The braiding process simulation model takes the machine control and process parameters as inputs and generates the braiding structure as an output. As shown in Figure 3a and Supplementary Video S1 (see Supplementary Materials), the simulation model on the macroscale contains the yarns and nonlinear springs. One end of the yarns is coupled to the take-up point, and the other end is connected to a spring element with constant tension force. Figure 2a displays the movement paths of the carriers applied to these control points. The take-up point moves upward at a constant speed corresponding to the carriers’ speed. The yarn-to-yarn friction coefficients for parallel- and perpendicular-to-yarn orientations were experimentally measured to be 0.26 and 0.46 [23]. However, the inter-yarn friction coefficient cannot be changed according to the yarn’s cross angle in the braiding simulation. Therefore, the interactions between yarns are defined using the Coulomb friction model with a friction coefficient of 0.36, which is the average of two directions. The braiding process is a quasi-static process, and a stable fabric structure requires multiple braiding cycles. It is essential to speed up the model calculation without compromising the computational accuracy. Reducing the modulus decreases the wave velocity in the material, facilitating a faster analysis speed. Comparative calculations show that setting the yarn’s modulus as 20,000 MPa is adequate to avoid stretching the yarn and the contact penetration.

Since a yarn consists of a substantial number of fibers, the bending modulus of yarns is much lower than the value calculated using Young’s modulus in the fiber direction. The primary purpose of the braiding process simulation is to achieve the topology structure of the preform. Using truss elements (T3D2) that neglect the bending stiffness approximates the actual state of yarns in the braiding process [22,28]. In Section 3.3, the yarn section deformation due to squeezing will be computed. The comparative analysis results show that the element size setting at half of the yarn radius can achieve minor penetration. Meanwhile, the contact control parameters were set to avoid a reduction in the contact thickness owing to the small element-size-to-truss-radius ratio [29]. The primary process parameters of the 3D4D braiding process in Table 1 and the trajectory of each yarn carrier were parameterized to facilitate modeling. Then, the corresponding Abaqus input file was automatically generated through the user-written Python script.

**Figure 4:**
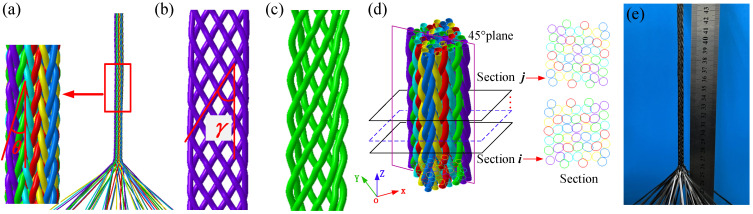
The braiding simulation results: (**a**) the simulation preform, (**b**) the yarn path in group 1, (**c**) the yarn path in group 5, (**d**) the pitch length calculating method, and (**e**) the test specimen.

**Figure 5:**
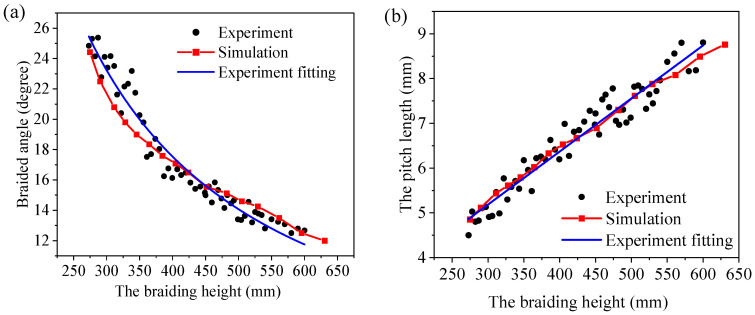
Comparison of simulation and experimental results: (**a**) the braiding angle and (**b**) the pitch length.

Figure 4a shows that the simulation result of the braiding preform is stable and the length of each pitch is also equal. The yarns in the same group have the same path, as shown in Figure 4b,c. Figure 4e shows the test specimen. In addition, another Python script was written to achieve the pitch length and braiding angle along the braiding direction. The center curve of every yarn was drawn through Abaqus’s application programming interface method. These curves were cut by sequential planes perpendicular to the braiding direction with a small resolution distance. The matching degree of two pair intersections was computed using the CDIST function in SciPy [30]. As shown in Figure 4d, the pitch length corresponding to one machine cycle was found when the error in the matching degree between two sections reached the minimum value. The interior braiding angle γ calculated from the yarn path in group 1 (Figure 5b) is the angle between the oblique line and the Z axis (the braiding direction) in the 45° plane. The surface braiding angle θ calculated from the yarn path in group 5 is the angle between the oblique line and the Z axis in the XOZ plane.

It is well known that the braiding process parameters must match each other to obtain a consistent fabric structure. For example, the fixed height of the interweaving point and the distance between two yarn carriers determine the braiding angle. The take-up speed and the time for each carrier to move one step decided the interweaving point of the braiding process. Therefore, the model with the take-up velocity as zero was implemented to investigate the relationship between braiding angle and interweaving point height. Despite the fluctuations in the braiding angle and height measurements of the experimental specimens, the simulation results are in good agreement with the experimental results, as shown in Figure 5. It is evident that the relationship between braiding angle and interweaving height is nonlinear, and the angle decreases as the height increases (Figure 5a). In addition, pitch length is positively correlated with braiding interweaving height (Figure 5b). The take-up machine moves up one pitch length in one braiding cycle to obtain a consistent preform. Meanwhile, it is necessary to match the pitch length and the interweaving point height by controlling the speed of the take-up device.

### 3.2. Sub-Yarn Discretization

The yarn center curves are obtained from the simulation method described in Section 3.1. However, the yarn section is circular due to the non-deformability of the truss element section, which is different from the actual yarn. To simulate the yarn squeezing deformation, the single yarn was discretized as several virtual sub-yarns whose fiber volume fraction is equal to the actual yarn, which was 65.3% in T700 12K [31]. The diameter of the sub-yarns denoted as $D_{vf}$ is determined by the equation as follows:

(2) $$A_{real}V_{fvf}=n_{vf}\frac{\pi }{4}D_{vf}^{2}$$

where $A_{real}$ and $V_{fvf}$ are the real yarn area and the fiber volume fraction, respectively, and $n_{vf}$ and $D_{vf}$ are the number and diameter of the virtual sub-yarns, respectively.

**Figure 6:**
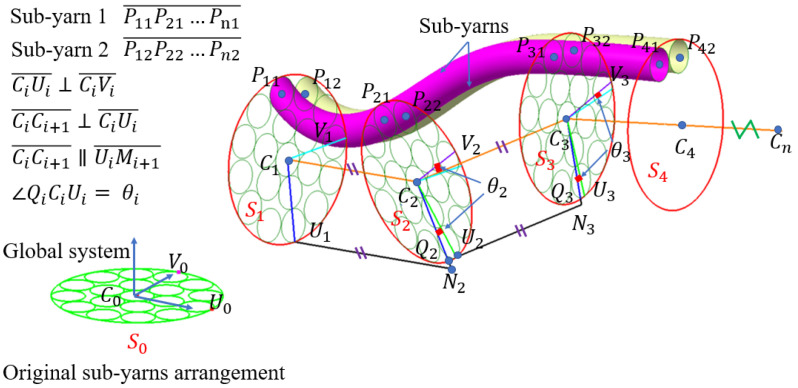
Illustration of the yarn discretization algorithm.

The sub-yarn discretization is implemented in a custom Python script. Figure 6 shows the schematic of the sub-yarn discretization process. $\bar{C_{1}C_{2}\ldots C_{n}}$ is the centerline of the yarn, the coordinates of which are extracted from the simulation result of the 3D braiding process in Section 3.1. $S_{0},S_{1},\ldots S_{n}$ are the sections vertical to the centerline of the yarns. $U_{1}$ is a point at the circumference of section $S_{1}$, and vector $\vec{C_{1}V_{1}}$ is achieved by rotating vector $\vec{C_{1}U_{1}}$ counterclockwise 90° with $\vec{C_{1}C_{2}}$ as the axis. Then, a line parallel to vector $\vec{C_{1}C_{2}}$ is drawn from point $U_{1}$, which intersects with $S_{2}$ at a point denoted as $N_{2}$. $\bar{C_{2}N_{2}}$ intersects with section $S_{2}$ at $Q_{2}$. If the yarn is twisted, the corresponding rotating angle $\theta_{i}$ can be calculated by the formula below.

(3) $$\theta_{i}=\frac{2\pi \phi \|\vec{C_{i}C_{i+1}}\|}{1000}$$

where ϕ is the twist of the yarn. The points $C_{i}$, $U_{i}$, and $V_{i}$ can be determined in sequence by repeating the earlier process. Then, the coordinate transform relationship $M_{i}$ from $C_{0}U_{0}V_{0}$ to $C_{i}U_{i}V_{i}$ is as follows:

(4) $$M_{i}\cdot \left[\begin{array}{ccc} x_{c_{0}} & x_{u_{0}} & x_{v_{0}}\\ y_{c_{0}} & y_{u_{0}} & y_{v_{0}}\\ z_{c_{0}} & z_{u_{0}} & z_{v_{0}} \end{array}\right]=\left[\begin{array}{ccc} x_{c_{i}} & x_{u_{i}} & x_{v_{i}}\\ y_{c_{i}} & y_{u_{i}} & y_{v_{i}}\\ z_{c_{i}} & z_{u_{i}} & z_{v_{i}} \end{array}\right]$$

**Figure 7:**
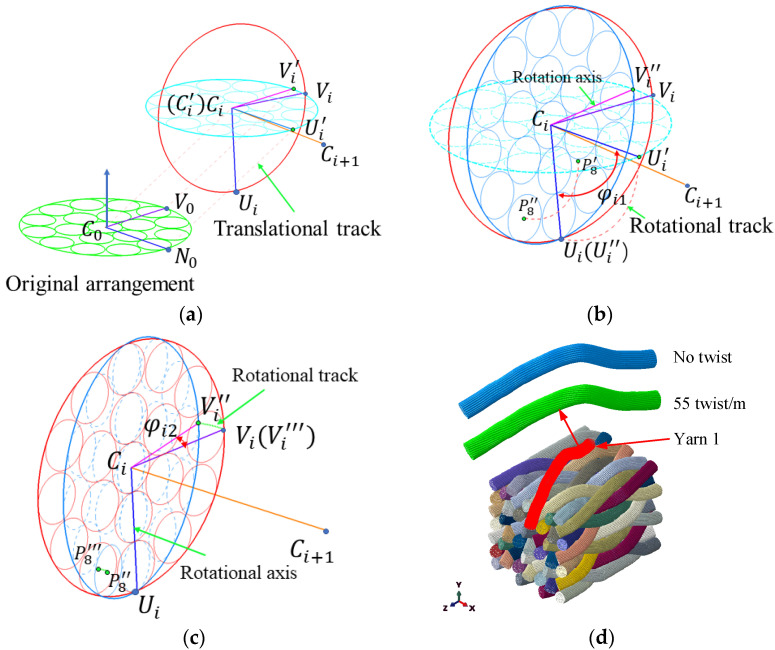
Illustration of the yarn coordinate transformation algorithm: (**a**) Step 1, involving the translational transformation, (**b**) Step 2, involving the first rotation transformation, and (**c**) Step 3, involving the second rotation transformation; (**d**) the result of the discretization of the yarns.

However, in some exceptional cases, the coordinate matrix does not have an inverse matrix, making it impossible to calculate the transformation matrix $M_{i}$. A new method to handle all situations was proposed, including one translational transformation and two rotational transformations. The method includes the following steps:

- The translational transformation was conducted to ensure that points $C_{0}$ and $C_{i}$ coincide, as shown in Figure 7a. The coordinates of points $C_{0}$ and $C_{i}$ are denoted as ${\left(x_{c_{0}},y_{c_{0}},z_{c_{0}}\right)}^{T}$ and ${\left(x_{c_{i}},y_{c_{i}},z_{c_{i}}\right)}^{T}$, respectively. The vector $\vec{C_{0}C_{i}}$ is denoted as ${\left(x_{t},y_{t},z_{t}\right)}^{T}$. The translation matrix $M_{i1}$ is as follows:
  (5) $$\left[\begin{array}{c} x_{c_{i}}\\ y_{c_{i}}\\ z_{c_{i}}\\ 1 \end{array}\right]=\left[\begin{array}{cccc} 1 & 0 & 0 & x_{t}\\ 0 & 1 & 0 & y_{t}\\ 0 & 0 & 1 & z_{t}\\ 0 & 0 & 0 & 1 \end{array}\right]\left[\begin{array}{c} x_{c_{0}}\\ y_{c_{0}}\\ z_{c_{0}}\\ 1 \end{array}\right]=M_{i1}\left[\begin{array}{c} x_{c_{0}}\\ y_{c_{0}}\\ z_{c_{0}}\\ 1 \end{array}\right]$$
- The rotational transformation was deployed to ensure that points $U_{i}^{\prime }$ and $U_{i}$ coincide. The rotation axis $\vec{C_{i}V_{i}^{\prime }}$ is determined by the cross product of vectors $\vec{C_{i}U_{i}^{\prime }}$ and $\vec{C_{i}U_{i}}$, as shown in Figure 7b. The unit vector $\vec{n}$ of the rotation direction and the rotation angle $\varphi_{i1}$ are calculated as follows:
  (6) $$\vec{n}=\frac{\vec{C_{i}U_{i}}\times \vec{C_{i}U_{i}^{\prime }}}{\left\|\vec{C_{i}U_{i}}\times \vec{C_{i}U_{i}^{\prime }}\right\|}={\left(n_{1},n_{2},n_{3}\right)}^{T}$$
  (7) $$\varphi_{i1}=arccos(\frac{\vec{C_{i}U_{i}^{\prime }}\cdot \vec{C_{i}U_{i}}}{\left\|\vec{C_{i}U_{i}^{\prime }}\|\|\vec{C_{i}U_{i}}\right\|})$$
  The rotation matrix $M_{i2}$ is presented in Equation (8), where $c=cos\left(\varphi_{i1}\right)$ and $s=sin\left(\varphi_{i1}\right)$. The blue lines (Figure 7b) are sub-yarns after rotation.
  (8) $$M_{i2}=\left[\begin{array}{cccc} n_{1}^{2}\left(1-c\right)+c & n_{1}n_{2}\left(1-c\right)-n_{3}s & n_{1}n_{3}\left(1-c\right)+n_{2}s & 0\\ n_{1}n_{2}\left(1-c\right)+n_{3}s & n_{2}^{2}\left(1-c\right)+c & n_{2}n_{3}\left(1-c\right)-n_{1}s & 0\\ n_{1}n_{3}\left(1-c\right)-n_{2}s & n_{2}n_{3}\left(1-c\right)+n_{1}s & n_{3}^{2}\left(1-c\right)+c & 0\\ 0 & 0 & 0 & 1 \end{array}\right]$$
- Taking the vector $\vec{C_{i}U_{i}}$ as the axis, the angle $\varphi_{i2}$ between $\vec{C_{i}V_{i}^{\prime \prime }}$ and $\vec{C_{i}V_{i}}$ is rotated so that point $V_{i}^{\prime \prime }$ coincides with point $V_{i}$ and the corresponding rotation matrix $M_{i3}$ is obtained, as illustrated in Figure 7c. After the three transformations, points $C_{0}$, $U_{0}$, and $V_{0}$ coincide at points $C_{i}$, $U_{i}$, and $V_{i}$, respectively.

All nodes in the original sub-yarn layout can be transferred to the target location by the following expression:

(9) $$\left[\begin{array}{c} x_{p_{i,j}}\\ y_{p_{i,j}}\\ z_{p_{i,j}}\\ 1 \end{array}\right]=M_{i3}M_{i2}M_{i1}\left[\begin{array}{c} x_{p_{0,j}}\\ x_{p_{0,j}}\\ x_{p_{0,j}}\\ 1 \end{array}\right]\;\;\;\;\;\;\;\;\;\;\;\;\;\;\;\;\;\;\;\;\;\;where\;\{\begin{array}{c} i=0,1,2\cdots m\\ j=0,1,2\cdots n \end{array}$$

where m refers to the section number and n refers to the number of sub-yarns. The subscripts i and j represent the section number and the sub-yarn number, respectively. The coordinate of the jth point on the ith section is $\left(x_{p_{i,j}},y_{p_{i,j}},z_{p_{i,j}}\right)$.

Figure 7d shows the discretization results of the yarns with 61 chains of sub-yarns. There is no interpenetration among the yarn sections. It is also able to achieve twisted yarn through this method.

### 3.3. Sub-Yarn Deformation on the Mesoscale

**Figure 8:**
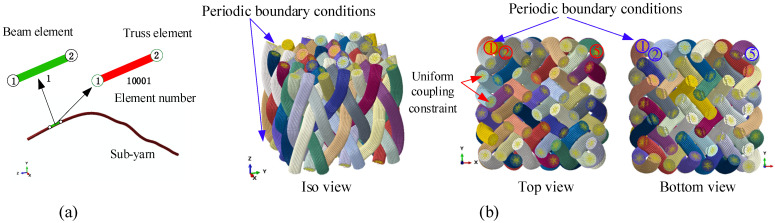
The FEA model of the yarn deformation simulation: (**a**) the hybrid element and (**b**) the periodic boundary condition.

The extrusion deformation among yarns is the dominant form in sub-yarn shrinkage simulations. The section shape of the yarn is mainly influenced by fiber bending stiffness that affects the realignment resistances, so the bending stiffness of virtual sub-yarns cannot be neglected. A new mesh technique was used to consider the appropriate bending stiffness of yarns by overlaying the truss element (T3D2) with beam elements (B31) with the coincident nodes [32], as shown in Figure 8a. The hybrid element, where the truss element determines the tensile stiffness (fiber orientation), while the beam element represents the bending property of the sub-yarns with a lower Young’s modulus, can simulate the real mechanical property. The beam radius is the same as the truss’s radius. The bending stiffness of the beam elements is calculated as follows:

(10) $$n_{vf}E_{beam}I_{vf,beam}=n_{rf}E_{rf}I_{rf}$$

where $I=0.25\pi r^{4}$ is the second moment of inertia and r is the radius of fiber; $n_{vf}$ and $n_{rf}$ denote the number of sub-yarns and actual fiber, respectively; $E_{beam}$ and $E_{rf}$ denote the Young’s modulus of the beam element and the actual fiber, respectively.

The 3D4D braiding composite is periodic in the braiding direction. Taking into account the edge effects and the computing cost, two pitches in a stable braiding state were chosen for sub-yarn deformation compression analysis. The end nodes of every bundle of sub-yarns were coupled to a control point using a uniform coupling constraint. The periodic boundary conditions (PBC) were applied to the two corresponding control points (Figure 8b). The linear constraint equations (*Equation) in Abaqus were adopted to realize the displacement constraint of the control points, and the formula was as follows:

(11) $$u_{i,a}=u_{i,b}\;\;\;\;\;\;\;where\;i=1,2\cdots 6,\;\;\;\;a,b=1,2\cdots N$$

where $u_{i}$ refers to the displacement of the freedom i, subscripts a and b refer to the control point numbers and N refers to the quantity of yarn.

To achieve a realistic braiding fabric structure, the temperature dropping load was imposed on all elements to ensure that the yarns squeezed with each other, which was equivalent to exerting tension force during the braiding process. The temperature drop stopped until the average stress of all elements was equal to the tension stress produced by the tension force of the yarn carrier. The following equation determines the average stress:

(12) $$s_{avg}=\overset{N}{\underset{i=0}{\sum }}\left(s_{11,i}^{beam}+S_{11,i}^{truss}\right)/N$$

where $S_{avg}$ is the average stress; $s_{11,i}^{beam}$ and $S_{11,i}^{truss}$ are the tension stress of the beam and truss elements, respectively; and N denotes the total element number of beam elements.

**Figure 9:**
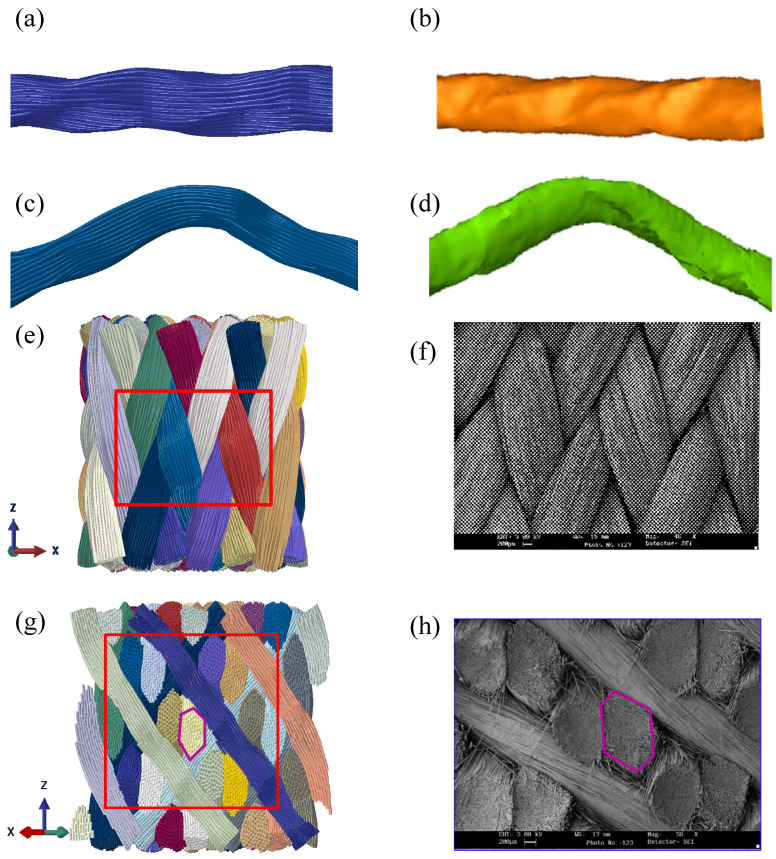
Comparison of the virtual structure and the experimental results: (**a**) the simulation interior yarn, (**b**) the micro-CT interior yarn [13]. Reprinted with permission from [13], Copyright 2017, Springer Science Business Media B.V., (**c**) the simulation surface yarn, (**d**) the micro-CT surface yarn [13]. Reprinted with permission from [13], Copyright 2017, Springer Science Business Media B.V, (**e**) the surface in the simulation, (**f**) the surface in the experiment [33]. Reprinted with permission from [33], Copyright 2012 Elsevier Ltd., (**g**) the 45° cross-section in simulation, and (**h**) the 45° cross-section in the experiment [34]. Reprinted with permission from [34], Copyright 2013 Elsevier Ltd.

The virtual yarn structure and the experiment results were compared, as shown in Figure 9. The localized distortions are observed, and the fibers are no longer parallel to the yarn centerline due to the lateral compression of adjacent yarns. The axes of yarns are spatial curves, and the section deformations are complicated. The shape of the interior yarn in simulation (Figure 9a) agrees well with the micro-CT interior yarn (Figure 9b); The surface yarn in simulation (Figure 9c) has good consistency with the micro-CT result (Figure 9d). Furthermore, Figure 9e–h compares the surface shape and the 45° cross-section of the preform between the simulation result and the SEM micrograph. The cross-sectional shape of yarns and the arrangement of braiding yarns are similar to the experimental observations. The comparison results confirm the validity of the numerical method. The Animation of the sub-yarn deformation is shown in Supplementary Video S2 (see Supplementary Materials).

### 3.4. Mesostructure Reconstruction

**Figure 10:**
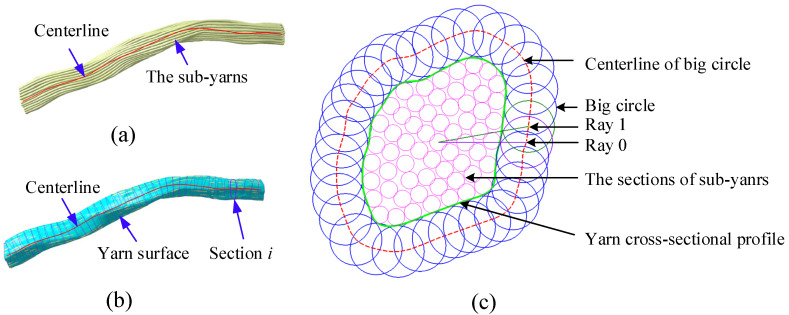
Illustration of the yarn geometry extraction process: (**a**) the fiber-level geometry from yarn deformation simulation, (**b**) the yarn geometry, and (**c**) the process of section profile extraction.

Most braiding composite FEA analyses are based on the yarn level. Fiber-level geometry needs to be transferred to yarn-level geometry. The center fiber (Figure 10a) determines the sequential cross-sections’ geometric centers and normals. The detailed extraction process of the yarn geometry involves three steps:

- The evenly distributed rays are drawn along the circumferential direction, with the center of the section as the starting point, as shown in Figure 10c. The center of the big circle moves outward from the center in steps of 0.001 mm on the ray until it does not intersect all sub-yarn sections. The big circle’s radius is set as 3–5 times the fiber radius to achieve a smooth and precise profile.
- The centers of all big circles are connected to form a curve. Then, the curve is offset by the radius of the big circle inward to obtain a curve that surrounds all fibers, that is, the cross-sectional profile of the yarn.
- All the cross-sectional profiles are joined to a multi-section surface, as shown in Figure 10b. These procedures are implemented with user-written Python scripts to obtain the coordinate sub-yarn nodes from the FEA result and generate realistic yarn geometry in Catia software.

**Figure 11:**
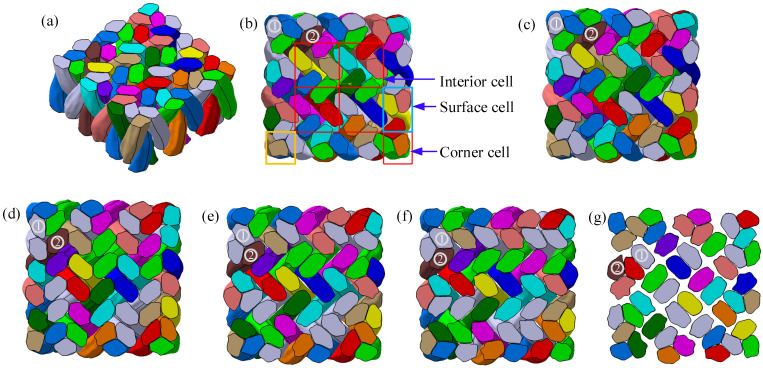
The sequential sections of a 3D4D braiding composite: (**a**) the iso view, (**b**) the top view, (**c**) the h/5 section, (**d**) the 2h/5 section, (**e**) the 3h/5 section, (**f**) the 4h/5 section, and (**g**) the 5h/5 section.

Figure 11a shows the extracted structure in one pitch. All yarns are anti-symmetric with respect to the center of the preform. In the [6×6] braiding preform, there are four interior cells, eight surface cells, and four corner cells, as presented in Figure 11b. Furthermore, the most detailed description of the mesostructure can be procured by comparing the changes in the sequential cross-sections of the preform along the braiding direction [9]. As shown in Figure 11b–g, four sections in one pitch are cut along the braiding direction, with equal distance between each section, as h/5. It is obvious that the yarn layouts of Figure 11b,f are almost identical, indicating that the mesostructure of 3D4D braiding composites is repeated for every pitch.

The essential purpose of our work is to obtain accurate RVEs for subsequent mechanical calculations. The solid structure models of three types of RVEs, including the interior, surface, and corner cells, are periodic, as shown in Figure 4f–h. Thus, it is conducive to generate the periodic mesh and impose the periodic conditions in subsequent mechanical calculations.

## 4. Parametric Study

The parameter study is performed to verify the accuracy and convergence performance of the finite element model. How to set the number of virtual sub-yarns to balance the calculation accuracy and efficiency, and the effects of sub-yarn stiffness, tension force, and yarn twist on the structure need further study.

### 4.1. Geometrical Convergence

**Figure 12:**
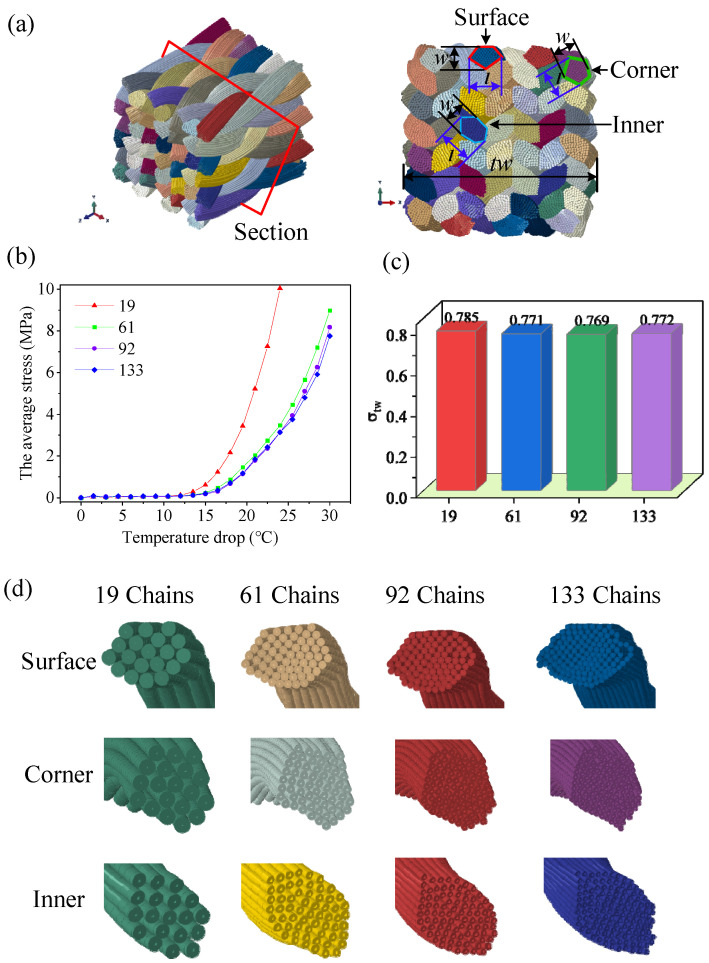
Geometrical convergence of the simulation models: (**a**) the cross-section of the preform after shrinkage, (**b**) the average stress curve of all virtual fibers, (**c**) the ratio of total width before and after deformation, and (**d**) the geometrical convergence of the cross-section shapes against the number of sub-yarns.

Owing to the enormous computational effort required, it is impossible to model each fiber as a single chain of beams. A parametric study of the sub-yarn number must be performed to balance the discrete precision of yarn cross-section deformation and the computation cost. Four models, discretized as the 19, 61, 92, and 133 chains of sub-yarns per yarn with the same yarn bending stiffness, were simulated to study the convergence of the model to reproduce an accurate braid structure. Figure 12a shows the result after the temperature drop process, and the cross-section in the middle was chosen to observe the deformation. Figure 12b illustrates the average stress representing the tension force of yarns versus the temperature drop value during the deformation. It is noted that each curve could be divided into three parts: linear, non-linear, and linear. The curves in the first part are flat, with low tension stress, and a kinematic response is evident where the virtual fibers rearrange and fill the gaps. The second parts show non-linear behavior, where the fibers reorient with increasing contact. In the third part, the average stress increases rapidly when fibers are sufficiently rearranged, resulting in a higher fiber volume fraction.

A higher number of sub-yarns leads to greater freedom of deformation of the yarn, so the average stress rises more slowly. The curve of 92 sub-yarns almost coincides with the curve of 133 sub-yarns, demonstrating that the model converges using 92 sub-yarns. Meanwhile, the ratio of total width change ($\sigma_{tw}=tw/tw_{0}$), representing the overall degree of deformation when the average stresses are equal to 1 MPa, is plotted in Figure 12c, showing that the number of sub-yarns has almost no effect on the overall deformation. Furthermore, three types of yarns, surface, corner, and interior, are compared in Figure 12d. The surface and corner yarns change from ellipse to pentagon, while the interior yarns change from ellipse to hexagonal. The cross-sectional shape of 92 sub-yarns is the same as that of 133 sub-yarns, and there is no penetration between the sub-yarns. Hereafter, 92 sub-yarns were used to model the yarn.

### 4.2. The Bending Stiffness

**Figure 13:**
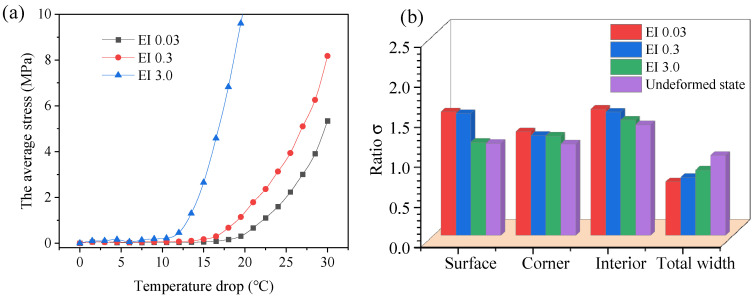
The effect of bending stiffness: (**a**) diagram of the average stress against temperature drop and (**b**) comparison of yarn deformation ratio and total width variation.

The changes in fiber bending stiffness by imposing beam elements of different bending stiffness affect yarn spreading. Figure 13a illustrates the average stress versus temperature drop values, which are plotted for three different moduli of the beam elements, of 133.6 MPa, 1336 MPa (the baseline, ${\left(EI\right)}_{yarn}=0.3$), and 13,360 MPa, with the same friction coefficient and tensile stiffness of the truss elements. The results show that the average stress rises faster as the bending modulus increases, indicating that the bending modulus cannot be neglected. Moreover, the degree of cross-sectional deformation is characterized by the ratio (σ=l/w) of the length (*l*) to the width (*w*) at a specific tension force (see Figure 12a). A smaller bending modulus of yarns, whether surface yarn, corner yarn, or interior yarn, leads to lower resistance of the yarn spreading to transverse deformation (Figure 13b), resulting in a larger ratio σ. The total width variation ($\sigma_{tw}$) increases as the bending stiffness decreases at the same yarn tension force, resulting in a higher yarn volume fraction.

### 4.3. The Tension Force of Carriers and the Twisting of Yarns

**Figure 14:**
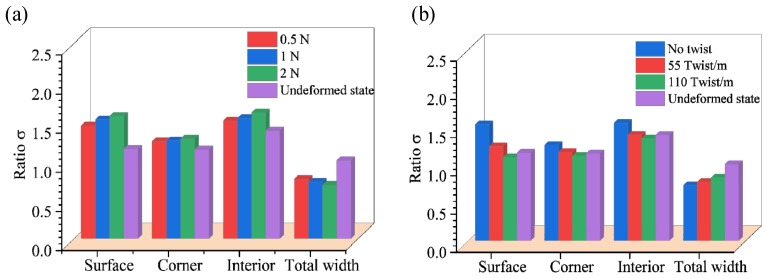
Diagram of the yarn deformation ratio and the total width variation. (**a**) with 0.5 N, 1 N, and 2 N tension force, respectively, and (**b**) with the no twist, 55 twist/m and 110 twist/m, respectively.

The 3D braiding process is fully automated and does not require manual jamming. The yarn carriers can control the yarn tension force at a constant value by retracting or releasing the yarns. Three simulations were performed with an increasing tension force of 0.5 N, 1 N, and 3 N, respectively, corresponding to the average stress of 0.98 MPa, 1.96 MPa, and 2.94 MPa. As shown in Figure 14a, the degree of yarn squeezing increases with the tensioning force and vice versa for the total width.

The twisted yarn has a higher resistance to cross-section deformation than the straight yarn [35]. Using the same tension force, three models, containing 55 twist/m, 110 twist/m, and no twist, were generated, the results of which are compared in Figure 14b. The ratio of section deformation degree in the twist condition is smaller than that in the no-twist situation, while the total width exhibits the reverse relationship. The twisting process increases the lateral stiffness of the yarn, and a suitable twist can reduce fiber pilling during the braiding process. However, too much twist increases the shear force among the fiber filaments, which is not conducive to the overall tensile strength. Therefore, it is necessary to consider yarn twisting in simulation.

## 5. Application

**Figure 15:**
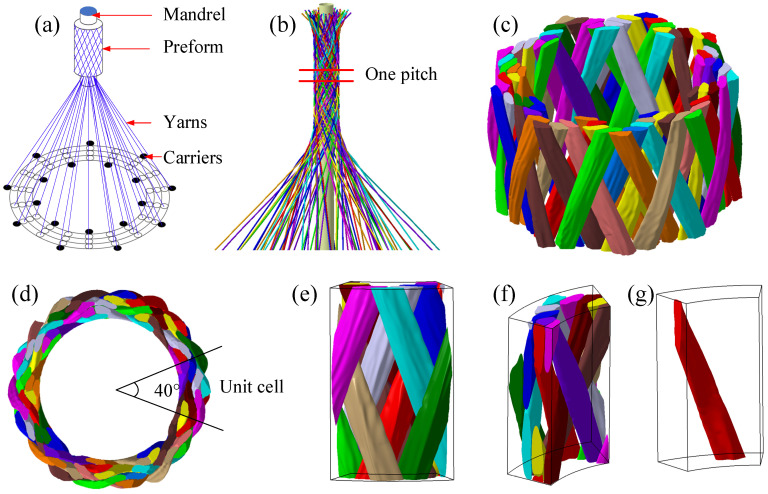
Simulation result of the 3D four-directional cylindrical braiding composite: (**a**) schematic of a cylindrical braiding machine, (**b**) the braiding process simulation result, (**c**) the mesostructure after deformation simulation, (**d**) the top view of one pitch, (**e**) the outside view of an RVE, (**f**) the inside view of an RVE, and (**g**) the deformational yarn.

The 3D braiding composite shafts are widely used in aerospace, wind power, and automotive transmission systems. These shafts can be integrally braided using the cylindrical braiding machine shown in Figure 15a [36,37]. The yarn arrangement consists of three layers in the radial direction, 18 columns in the circumferential direction, and the side yarns. The yarn carriers move alternately in one position in radial and circumferential directions. After four steps, the take-up device lifts one pitch height upward correspondingly. Figure 15b shows the simulation result of the braiding process, and Figure 15c shows the result of yarn deformation on the mesoscale. The yarns are arranged periodically in the circumferential direction. Figure 15d–f shows the RVE, and the yarn cross-section changes from a circle to a complex shape. Therefore, the application in the 3D cylindrical braiding process demonstrates the versatility of the simulation methodology.

## 6. Conclusions

The present research developed a multiscale simulation methodology based on the virtual sub-yarns to establish accurate RVEs. The simulation results were compared with the micro-CT and SEM structures in the literature. A parametric study was conducted to validate the model. The multiscale method yields accurate results with a relatively small computational cost. Furthermore, the method could give some predictive guidance on the process design in the early stage. The following conclusions can be drawn:

(1) The braiding angle decreases as the height of the braiding point increases and vice versa for pitch length.
(2) To achieve a realistic interior cell, surface cell, and corner cell, a temperature drop simulation with hybrid elements was conducted to make the yarn cross-section deformable. The simulation result shows good agreement with the experiment.
(3) The parametric study shows that the use of 92 sub-yarns represents the cross-sectional deformation well. A smaller bending stiffness leads to a larger total width variation, resulting in a higher yarn volume fraction at the same tension force. The yarn deformation degree is positively related to the tension force of carriers. Finally, the twisted yarn has a higher resistance to cross-sectional deformation than the straight yarn.
(4) The simulation methodology presented here is a versatile tool that can study the relationship between the different braiding processes and the mesostructure of the preform.

This paper proposes a numerical method to obtain the accurate RVEs. Future works will integrate the source codes (see Supplementary Materials) into Abaqus Plug-ins to further accelerate engineers’ research and development work.

## Figures and Tables

**Table 1:** The main process parameters of the 3D4D braiding process.

Take-up distance of the preform in one machine cycle h
Distance between every two adjacent carriers *D*
The tension force of carrier *F*
The braiding interweaving height *H*

**Table 2:** Material properties of the yarn.

Density (g/cm^3^)	1.8
Number of fibers per yarn	12,000
Modulus of elasticity (GPa)	230
Coefficient of friction among yarns	0.36

## Data Availability

Data are contained within the article.

## Supplementary Materials

The following are available online at https://www.mdpi.com/article/10.3390/polym14194210/s1. Video S1: the simulation results of the 3D4D braiding process, Video S2: the sub-yarn deformation, and the source codes of our workflow.
