# Supplementary material for: A Methodology to Obtain the Accurate RVEs by a Multiscale Numerical Simulation of the 3D Braiding Process
Source: Polymers (Basel). 2022 Oct 7;14(19):4210. doi: 10.3390/polym14194210 (PMC9572426; doi:10.3390/polym14194210)
Supplement: Supplementary file 1 [file polymers-14-04210-s001.zip › Source codes/The user-written scripts readme.pdf]

Python version: 2.7

Abaqus version: 2020

Catia version: R2018

The present research developed a multiscale simulation methodology based on the virtual sub-yarns to establish accurate RVEs. We developed several user-written scripts to accelerate the modeling process. All the scripts are listed below:

Table S1: the list of Python scripts

	Function description	Input	Output	Section in article
C3D4_braiding.py	Creating the parametric model for the braiding process simulation	The basic braiding machine parameter, the carrier arrangement, the trace of every carrier	Abaqus input file	Section 3.1
YarnCoordGet.py	Using Abaqus API method to obtain the coordinates of yarns from Abaqus result file	The Abaqus result file: C3d4.odb	The coordinates of yarn center line	Section 3.1
findPeriodLength.py	Achieving the pitch length and the braiding angle	The coordinates of yarn center line	The pitch length and angle	Section 3.1
yarnDiscrete_twist.py	Sub-yarn discretization	The coordinates of yarn center line, the pitch length	The Abaqus input file of Yarn Deformation on a Mesoscale	Section 3.2
catiaGeoRconstruction.py	Obtaining the surface info of every yarn and creating the geometry through Catia API method	The sub-yarn coordinate from yarn deformation simulation in section 3.3	The RVE geometry	Section 3.4
