# Supplementary figures and images for: A Methodology to Obtain the Accurate RVEs by a Multiscale Numerical Simulation of the 3D Braiding Process
Source: Polymers (Basel). 2022 Oct 7;14(19):4210. doi: 10.3390/polym14194210 (PMC9572426; doi:10.3390/polym14194210)

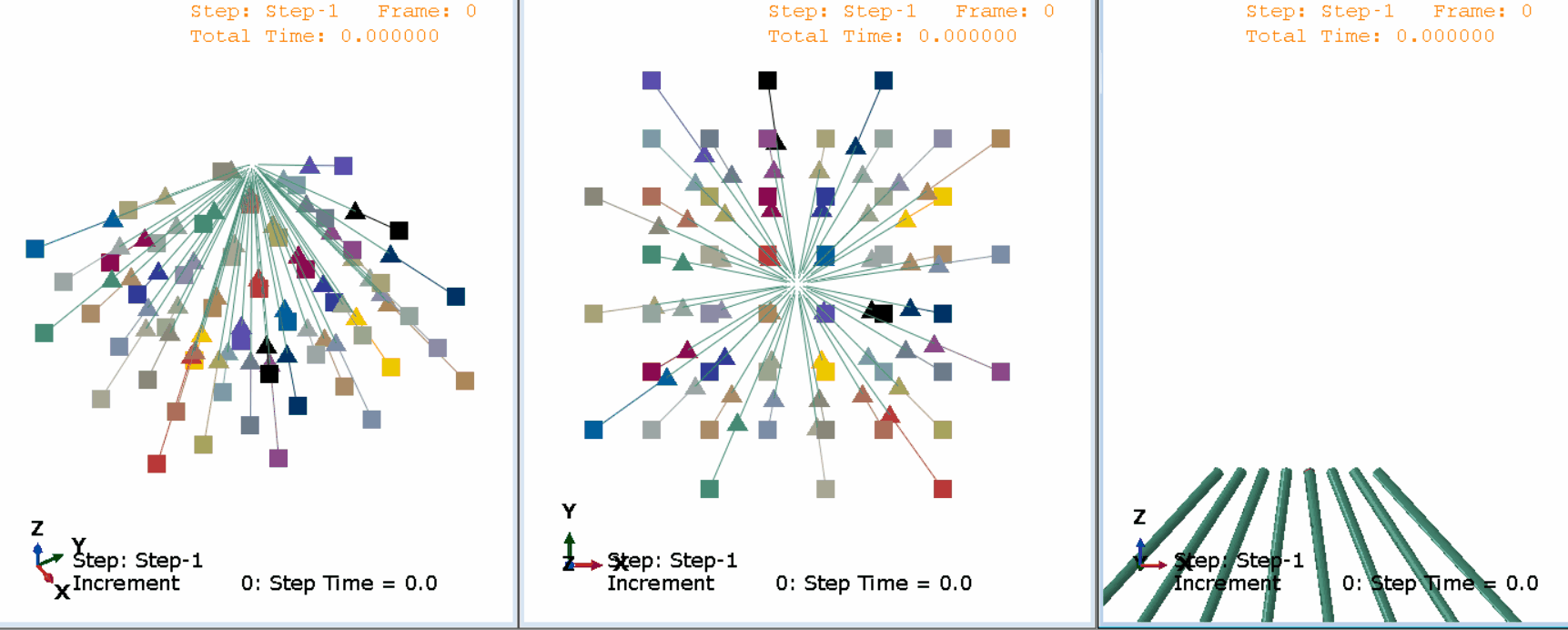

Supplement: Supplementary file 1 [file polymers-14-04210-s001.zip › Animation/Video S1.gif]

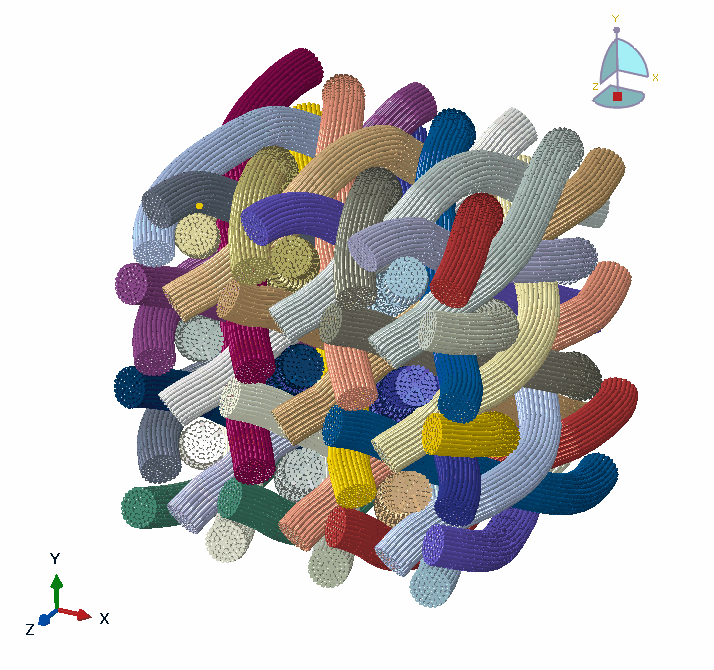

Supplement: Supplementary file 1 [file polymers-14-04210-s001.zip › Animation/Video S2.gif]
